# Available but inaccessible: patient experiences during the first 2 years of a primary care-based medical cannabis program at an academic medical center

**DOI:** 10.1186/s12954-023-00919-2

**Published:** 2024-01-02

**Authors:** Eloise W. Freitag, Yuval Zolotov, Jayabhargav Annam, Jaqueline Labins, Jaclyn M. Yamada, Syeda Masharab Jillani, Julia H. Arnsten, Deepika E. Slawek

**Affiliations:** 1 Harvard University, Cambridge, MA USA; 2 Division of General Internal Medicine, Montefiore Medical Center/Albert Einstein College of Medicine, 111 E 210th St, Bronx, NY 10467 USA

**Keywords:** Medical cannabis, Patient behaviors, Barriers, Harm reduction, Equitable access to care

## Abstract

**Background:**

Medical cannabis use and public acceptance in the United States have increased over the past 25 years. However, access to medical cannabis remains limited, particularly for underserved populations. To understand how patients experience medical cannabis accessibility, we measured medical cannabis use and barriers to use after medical cannabis certification in an urban safety-net academic medical center.

**Methods:**

We conducted a retrospective cohort study among patients seen in Montefiore’s Medical Cannabis Program (MMCP) from 2017 to 2019. Patient demographic and clinical characteristics, as well purchase history of medical cannabis, were extracted from electronic medical records. We also administered a phone questionnaire to a subset of patients to assess usage patterns, effectiveness, and barriers to medical cannabis use.

**Results:**

Among 562 patients who were newly certified for medical cannabis between 2017 and 2019, 45% purchased medical cannabis, while 55% did not. Patients who purchased medical cannabis were more likely to be white and have private insurance or Medicare. Unregulated cannabis use and current tobacco use were less common among those who purchased medical cannabis. In multivariable logistic regression analysis, unregulated cannabis use remained negatively associated with purchasing medical cannabis. Patients reported that affordability and dispensary accessibility were their main barriers to purchasing medical cannabis.

**Conclusion:**

Among patients certified for medical cannabis use, fewer than half purchased medical cannabis after certification. Improving access to medical cannabis is crucial for ensuring equitable access to regulated cannabis, and to reducing unregulated cannabis use.

## Introduction

Medical cannabis use and public acceptance of it in the United States (U.S) have increased over the past 25 years [1]. Cannabis remains a Schedule I substance under the U.S. Federal Government’s Controlled Substance Act [2], but the U.S Department of Justice leaves enforcement of cannabis laws up to individual states [3]. Medical cannabis is legal in 38 states and the District of Columbia as of April 2023 [4]. New York State (NYS) legalized and implemented a state-regulated medical cannabis program in 2016 [5].

From its inception through 2019, New York State’s (NYS) medical cannabis program was among the most heavily regulated medical cannabis programs in the U.S. [6]. It included state-based verification of cannabinoid content, testing for contaminants such as pesticides and other drugs, and requiring clinicians to complete training to certify patients. At that time, NYS’s medical cannabis program restricted medical cannabis products to only oil-based products that were delivered as vaporized oils, oil-based edibles, or sublingual tinctures. No whole flower products were available through the medical cannabis market prior to 2021 [7]. Soon after the NYS medical cannabis program was implemented, Montefiore Medical Center, a large urban safety-net academic medical center in the Bronx, NY developed and implemented Montefiore’s Medical Cannabis Program (MMCP) in its ambulatory care network [8].

Patients seek out medical cannabis for a range of indications, including but not limited to: severe or chronic pain, nausea or vomiting secondary to chemotherapy or other medical treatments, anxiety disorders, and insomnia [9]. Though medical cannabis programs make cannabis legally available to patients, they do not necessarily make it accessible to them. Prior research found that medical cannabis certifying providers and dispensaries are in areas that are more likely to be white and affluent [10]. Further, medical cannabis is not covered by any insurance, and must be paid for out of pocket (usually in cash).

While over 100,000 patients have been certified to use medical cannabis in New York State, little is known about patient behaviors after certification, including medical and unregulated cannabis use. Existing research is focused on characterizing patients by sociodemographic characteristics and primary conditions for which patients sought medical cannabis [11]. These studies do not show what happens after certification, including picking up medical cannabis products from dispensaries, and response to treatment in both patients who receive medical cannabis and those who do not. Further, they do not focus on patients accessing medical cannabis certification through coordinated medical cannabis programs housed within an academic medical center, such as the MMCP.

Among patients assessed in the first 2 years of the MMCP, when NYS’s medical cannabis program was most heavily regulated, we assessed use and barriers to use after medical cannabis certification with a retrospective chart review and phone questionnaires. We hypothesized that few patients who were certified in the MMCP would report purchasing medical cannabis more than once, and that baseline unregulated cannabis use would be associated with not purchasing medical cannabis.

## Methods

### Setting

The MMCP is a primary care-based program established in 2016 that operates in four clinics across the Bronx, NY, and has 13 certifying physicians [8]. Three out of four participating clinics are designated Federally Qualified Health Centers (FQHC), providing care to a wide range of patients, including those on Medicaid.

### Study design

We conducted a retrospective cohort study of patients seen in the MMCP from 2017 to 2019, with aims to: (1) understand the demographic and clinical characteristics of the population seeking medical cannabis at an urban, safety net, academic medical center, (2) examine the proportion of certified patients purchasing medical cannabis, and (3) examine associations between patient characteristics and medical cannabis purchases. We administered a phone questionnaire to a subset of the cohort. The study was reviewed and approved by the institutional review board at Albert Einstein College of Medicine (IRB# 2018-9274).

### Study population

Patients were seen at any of the four MMCP clinics by one of the 13 certifying physicians for medical cannabis certification. The patients included in the study: (1) presented to the clinics between August 2017 and August 2019, and (2) received their first New York state medical cannabis certification during that time frame. We conducted phone questionnaires from May 2017 to December 2020. Patients were eligible for the phone questionnaire if they were: (1) English speaking, and (2) certified no more than 1 year prior to the day of the survey, to limit risk of recall bias. Each participant who completed the phone questionnaire completed it one time. Patients were excluded if they were renewing their certifications or did not receive their certification.

### Data collection

We reviewed patient electronic medical records (EMR) for demographic characteristics, including age, sex, ethnicity (Hispanic, non-Hispanic White, and non-Hispanic Black), insurance status (Medicare, Medicaid, and private), and medical history. To review medical history, we assessed the “problem list” and the NYS medical cannabis certification form in each patient’s chart to determine all medical conditions and qualifying condition(s) for medical cannabis, respectively. We reviewed the progress note for the certification visit to determine prescription opioid use, non-medical cannabis use, and tobacco use. We reviewed the NY state prescription monitoring program (PMP) to determine purchase history of medical cannabis at dispensaries for patients who received medical cannabis certification at least three months prior, allowing for sufficient time to visit the dispensary.

We used a combination of chart review and extracted data from the NYS Medical Cannabis Data Management System to identify the patients that were certified for cannabis for the first time from 2017 to 2019. The NYS Medical Cannabis Data Management System is a state-managed database of all certifications completed by certifying providers in NYS. To understand whether patients picked up medical cannabis from a dispensary and factors associated with picking up medical cannabis, we administered a cross sectional phone questionnaire in a subset of the cohort. Our goal was to survey at least 100 patients and to include patients that had purchased medical cannabis and those who did not (according to the NYS PMP).

The phone questionnaire was a 5–8-min questionnaire administered by members of the research team (JL, JY, JA). Questions focused on whether participants purchased medical cannabis, route of administration of medical cannabis, symptoms patients were targeting, and response to treatment (see Appendix 1 for full survey). Symptom relief was assessed using a 0–10 Likert scale, with ‘0’ representing ‘not effective at all’ and ‘10’ representing ‘highly effective’. Those that never purchased medical cannabis were asked to identify the main reason for not purchasing medical cannabis by picking one of the following: price, ineffectiveness, convenience, perceived stigma, problems with registration, uncomfortable with the process, or other. All patients were additionally asked if they were using cannabis not purchased at a medical dispensary. Those that answered ‘yes’ were asked about the form of unregulated cannabis that they used and whether they obtained symptom relief.

### Data analysis

We analyzed patient demographic and clinical characteristics using descriptive statistics. To compare those who purchased medical cannabis with those who did not, we used t tests and chi-square tests. We used multivariable logistic regression to test how unregulated cannabis use was associated with medical cannabis purchase, adjusted for age, gender, insurance status, tobacco use, and prescription opioid use. We used a two-sided p threshold of 0.05, and report on adjusted odds ratios (aOR) and 95% confidence intervals (95% CI). In the cross sectional phone survey, we assessed patterns of use, perceived effectiveness, and access barriers. Analyses were conducted using SPSS (version 25).

## Results

### Demographic and clinical characteristics

We identified 562 patients who met criteria for inclusion in the cohort. Table 1 describes the demographic and clinical characteristics of our sample, stratified by whether patients purchased medical cannabis. The average age of the cohort was 51 years (standard deviation [SD] = 13.5). Sixty-two percent were female, 40.6% were Black, 48.3% identified as other race, and 45.9% identified as Hispanic. Nearly half of participants (47.5%) had health insurance through Medicaid. The most common reasons for seeking out medical cannabis were back pain (39.1%) and other musculoskeletal pain (33.9%). Concomitant substance use was common, with a mix of prescription, non-prescription, legal, and illegal substances: 34% of participants had documented tobacco use, 55.4% had documented unregulated cannabis use, and 40.9% were prescribed opioids.

**Table 1:** Demographic and clinical characteristics of patients certified for medical cannabis

	Total (n = 562)	Purchased medical cannabis n = 253 (45%)	Did not purchase n = 309 (55%)	*p* value*
Age (years), mean (SD)	50.6 (13.5)	51.79 (14.4)	49.60 (12.6)	0.06
Gender: female, n (%)	349 (62.1)	165 (65.2)	184 (59.5)	0.16
Race, n (%)				**< 0.01**
Black	215 (40.6)	94 (39.5)	121 (41.4)	
White	56 (10.6)	35 (14.7)	21 (7.2)	
Asian	3 (0.6)	3 (1.3)	0 (0)	
Other	256 (48.3)	106 (44.5)	150 (51.4)	
Ethnicity, n (%)				0.55
Hispanic	241 (45.9)	131 (55.5)	153 (52.9)	
Non-Hispanic	284 (54.1)	105 (44.5)	136 (47.1)	
Insurance, n (%)				< 0.01
Medicaid	265 (47.5)	101 (40.1)	164 (53.6)	
Medicare	193 (34.6)	96 (38.1)	97 (31.7)	
Private	100 (17.9)	55 (21.8)	45 (14.7)	
Qualifying condition, n (%)				
Pain (back)	220 (39.1)	97 (38.3)	123 (39.8)	0.72
Pain (other MSK)	190 (33.9)	88 (34.9)	102 (33)	0.63
Pain (fibromyalgia)	30 (5.3)	14 (5.5)	16 (5.2)	0.85
PTSD	17 (3)	9 (3.6)	8 (2.6)	0.50
Other	105 (18.7)	47 (18.5)	58 (18.7)	0.95
Substance use, n (%)				
Current prescription opioid use	230 (40.9)	115 (45.5)	115 (37.2)	**0.04**
Current unregulated cannabis use	297 (55.4)	111 (45.1)	186 (64.1)	**< 0.001**
Current tobacco use	187 (34.1)	60 (24.5)	127 (41.9)	**< 0.001**
Chronic medical conditions, n (%)				
Hypertension	234 (41.6)	104 (41.1)	130 (42.1)	0.81
Asthma	146 (26)	72 (28.5)	74 (23.9)	0.22
Depression	145 (25.8)	70 (27.7)	75 (24.3)	0.36
HIV	112 (19.9)	36 (14.2)	76 (24.6)	**< 0.01**
Arthritis	103 (18.3)	54 (21.3)	49 (15.9)	0.09
Obesity	63 (11.2)	47 (18.6)	48 (15.5)	0.33
Anxiety	89 (15.8)	44 (17.4)	45 (14.6)	0.36
Diabetes	63 (11.2)	29 (11.5)	34 (11)	0.86
Sickle cell	25 (4.4)	12 (4.7)	13 (4.2)	0.75

Statistically significant p-values are in bold

^*^Based on statistical tests comparing between participants who purchased medical cannabis and those who did not (independent t test for age, and chi-square test for all other variables)

MSK: Musculoskeletal; PTSD: Post-traumatic stress disorder

Of 562 certified patients, 45% purchased medical cannabis and 55% did not. Compared to those who did not purchase medical cannabis, those who purchased medical cannabis were more likely to be White race (14.7% vs. 7.2%) and less likely to identify as ‘other’ race (44.5% vs. 51.4%; *p* < 0.01). Patients who purchased medical cannabis were also more likely to have private insurance (21.8% vs. 14.7%) and Medicare (38.1% vs. 31.7%) and less likely to have Medicaid (40.1% vs. 53.6%) than patients who did not purchase medical cannabis (*p* < 0.01). Prescription opioid use was more common among patients who purchased medical cannabis (45.5% vs. 37.2%; *p* < 0.05) than those who did not. Unregulated cannabis use (45.1% vs. 64.1%; *p* < 0.001) and current tobacco use (24.5% vs. 41.9%; *p* < 0.001) were less common among those who purchased medical cannabis than those who did not. Finally, patients who were diagnosed with HIV were more likely to purchase medical cannabis than those who were not (14.2% vs 24.6%, *p* < 0.01).

In a multivariable logistic regression model (Table 2), unregulated cannabis use was negatively associated with purchasing medical cannabis when controlling for age, gender, race, insurance status, tobacco use, and prescription? Opioid use (aOR 0.63 [95% CI 0.42–0.95]; *p* = 0.02).

**Table 2:** Logistic regression predicting the likelihood of purchasing medical cannabis (n = 562)

Variable	aOR	*p* value	95% Confidence Interval	
			Lower	Upper
*Unregulated cannabis use*	0.63	0.02	0.42	0.95
Age	0.99	0.79	0.98	1.01
Gender	0.91	0.65	0.61	1.35
Race—Black	1.09	0.66	0.73	1.62
Race—White	1.97	0.04	1.03	3.77
Insurance—Medicare	1.18	0.49	0.73	1.89
Insurance—Private	1.56	0.08	0.93	2.62
Tobacco use	0.54	0.00	0.36	0.81
Opioids use	1.24	0.24	0.85	1.81

### Phone survey

We called 362 total patients from the cohort to conduct phone surveys. Of these, 119 met the inclusion criteria and completed the survey.

The characteristics of participants who completed the phone survey are presented in Table 3. Compared to the full cohort, a larger proportion of those who were surveyed were female (70.6% vs 62.1%, *p* = 0.03). Otherwise, those who were surveyed were similar to the full cohort. Most participants were seeking medical cannabis to manage pain (n = 101, 85.6%). Participants also reported seeking medical cannabis to manage anxiety (n = 7, 5.9%) or insomnia (n = 3, 2.5%). Overall, participants reported high perceived effectiveness for symptom relief (mean 6.9; SD = 2.92). Eighty-seven participants (73.1%) reported ever purchasing medical cannabis from a dispensary in NYS. Of them, only 63 (52.9%) purchased medical cannabis more than once. The most common reasons for not purchasing among those who did not purchase medical cannabis (n = 56, 47.1%) were price (n = 28, 50%), convenience of dispensary locations (n = 14, 25%), ineffectiveness (n = 6, 10.7%) and problems with registration (n = 4, 7.1%).

**Table 3:** Characteristics of participants who completed the phone survey (n = 119)

	n = 119
Age (years), mean (SD)	56.2 (12.1)
Gender: female, n (%)*	83 (69.7)
Main symptom targeted, n (%)	
Pain	101 (85.6)
Anxiety	7 (5.9)
Insomnia	3 (2.5)
Other	7 (5.9)
Ever purchased medical cannabis, n (%)	87 (73.1)
Purchased medical cannabis more than once, n (%)	63 (52.9)
Use of unregulated cannabis, n (%)	52 (43.7)
Reason for not purchasing medical cannabis (n = 56), n (%)	
Price	28 (50)
Convenience of dispensary locations	14 (25)
Ineffectiveness	6 (10.7)
Problems with registration	4 (7.1)
Perceived effectiveness of medical cannabis (n = 86), mean (SD)	6.9 (2.9)
Preferred route of administration of medical cannabis (n = 86), n (%)	
Oil	55 (64)
Vape	32 (37.2)
Capsule	24 (27.9)
Other	10 (11.8)

*Proportion of gender is significantly different than the whole cohort (n = 562)

## Discussion

In our analysis of patients who accessed certification for medical cannabis in an urban safety-net academic medical center, we found that though patients found medical cannabis to be effective for the management of their symptoms, there were many barriers to its use. Fewer than half of patients certified went on to purchase medical cannabis. Respondents acknowledged the effectiveness of cannabis in alleviating chronic pain. However, cost and dispensary location were barriers to purchasing medical cannabis.

We discovered that unregulated cannabis use was more prevalent among those who did not purchase medical cannabis. Some individuals may opt for unregulated sources, which are often cheaper than medical cannabis. It is possible that cost is a major barrier to switching from unregulated use to medical cannabis use. These findings highlight the importance of addressing affordability issues and improving accessibility to medical cannabis for individuals who can benefit from its pain-relieving properties.

There are ongoing efforts to understand the potential therapeutic benefits of medical cannabis in comparison with other treatment options [12, 13]. Access to medical cannabis offers a safer option than unregulated cannabis to patients who use it to manage clinical symptoms [14]. While federal legalization of medical cannabis has yet to occur, some have posited that increased access to regulated cannabis could reduce illicit cannabis markets and increase safe options for use [15].

Medical cannabis is legal in 38 states, and the movement to legalize is growing. Despite equal rates of use, people of color are arrested at higher rates for cannabis possession than white people [16]. Medical cannabis certification provides clinical justification for cannabis use for patients with symptoms that could potentially benefit from it, but access currently remains inadequate and inequitable. Previously identified barriers to access to medical cannabis include stigma, cost, and ease of access [17], and our findings reinforce these and extend them to patients in an urban safety-net hospital system. Systems changes are needed to ensure that medical cannabis is an affordable option, allowing for a switch from unregulated cannabis to medical cannabis in relevant patients. Our findings also reinforce the need for policies that ensure medical cannabis dispensaries are geographically accessible to all communities who may benefit from them.

We found that patients who purchased medical cannabis were more likely to be white and have private insurance. This points to a disparity in access to medical cannabis, impacting people of color and people with government insurance, which is often used as a proxy for low income. This is similar to findings from our group [10], and in other medical cannabis systems [18, 19]. Interestingly, patients with HIV were more likely to purchase medical cannabis. This could be because patients with HIV have more interaction with the healthcare system and have developed resources to understand complex healthcare system changes [20–22].

Our study and findings are novel. Two large studies reviewed state registry data of patients certified for medical cannabis, but they were unable to provide a complete picture for patient characteristics and medical conditions being treated due to lack of complete access to data [23, 24]. Other studies used data from a medical cannabis evaluation clinic system or by directly surveying established customers of dispensaries to understand customer’s relationship between their medical cannabis and their other medication use. However, these studies did not assess how accessible medical cannabis was to patients who had been certified for medical cannabis by a clinician. Our findings are also unique in that they are in the context of an urban safety-net academic medical center [25, 26].

Our study has limitations worth noting. Firstly, while the study is comprehensive in understanding a specific relevant geographic location (Bronx, NY) and a specific medical cannabis program (MMCP), this may limit the generalizability of the findings to other regions or programs. Additionally, the study only includes patients who sought medical cannabis certification at the MMCP clinics and received their first certification within a specific time frame. This may introduce selection bias and limit the representativeness of the patient population. Additionally, the study’s delineation between purchasers of medical cannabis and non-purchasers is complicated by the presence of individuals accessing cannabis through unregulated markets in both groups. Further, patients who did not purchase medical cannabis may have done so because they preferred whole flower cannabis, which was not available in dispensaries at the time of this survey [7]. Unfortunately, we did not ask whether patients preferred whole flower cannabis in our survey, and so cannot answer whether this was a reason for not purchasing medical cannabis. Furthermore, the study relies on self-reported data through phone questionnaires, which may be subject to recall bias and social desirability bias. Patients may not accurately recall or report their cannabis use, symptoms, or treatment response. The data from electronic medical records may also have limitations, such as missing or incomplete information, inconsistencies in documentation, or variations in record-keeping practices among healthcare providers. Finally, the study focuses on the first 2 years of the MMCP, with no information provided regarding long-term patient outcomes or behavior beyond this timeframe. This may be an interesting direction for future research.

Despite its limitations, this study also has many strengths. The longitudinal approach, covering a span of 2 years, allows for a more comprehensive understanding of patient behaviors and trends over time. Additionally, the multidimensional data collection, utilizing a combination of retrospective chart review, phone questionnaires, and data extraction from state-managed databases, provides a rich dataset for analysis. Furthermore, the study includes patients from a diverse urban population.

In conclusion, we found that certification did not guarantee access to medical cannabis in a group of patients accessing health care in the Bronx, NY. Systematic changes are needed to ensure that there is equitable access to medical cannabis across communities that have legalized it.

## Data Availability

The datasets used and/or analyzed during the current study are not publicly available due to concerns about protecting participants’ personal information, but de-identified data are available from the corresponding author on reasonable request.
